# Mural Lymphatic Endothelial Cells at CNS Borders: Endothelial Heterogeneity, Functional Specialization, and Cross-Species Perspectives

**DOI:** 10.3390/biology15171478

**Published:** 2026-09-01

**Authors:** Junxiang Xu, Yijin Chang, Jiahao Lu, Dong Liu, Changsheng Chen

**Affiliations:** School of Life Sciences, Co-Innovation Center of Neuroregeneration, Nantong University, Nantong 226019, China; jaysenxjx@stmail.ntu.edu.cn (J.X.);

**Keywords:** mural lymphatic endothelial cells, endothelial heterogeneity, cerebrovascular system, lymphatic endothelial cells, scavenger endothelial cells, single-cell transcriptomics

## Abstract

Recent advances in developmental biology and single-cell transcriptomics have revealed remarkable endothelial heterogeneity within the central nervous system, leading to the identification of specialized endothelial populations at brain border regions. Among these, mural lymphatic endothelial cells, also termed brain lymphatic endothelial cells in zebrafish, have emerged as a unique non-lumenized endothelial population with distinctive developmental and functional characteristics. In this review, we critically evaluate current evidence regarding the nomenclature, developmental origin, molecular identity, functional specialization, cross-species relationships, and disease relevance of mural lymphatic endothelial cell-related populations. Current studies demonstrate that zebrafish mural lymphatic endothelial cells depend on Vegfc–Vegfr3 signaling, express multiple lymphatic-associated markers, and exhibit exceptionally strong scavenger and endocytic activity while remaining closely associated with cerebral blood vessels rather than forming conventional lymphatic vessels. Recent transcriptomic analyses further identify these cells as a transcriptionally distinct endothelial population enriched in uptake, endocytosis, and intracellular trafficking pathways. We also distinguish established evidence from unresolved questions concerning developmental lineage, functional heterogeneity, and evolutionary relationships among related cell populations described in vertebrates. Collectively, current evidence supports mural lymphatic endothelial cells as a specialized endothelial population at central nervous system borders and highlights their importance for understanding endothelial heterogeneity, neurovascular homeostasis, and future studies of neurological and cerebrovascular diseases.

## Simple Summary

The brain depends on specialized cells to remove waste, maintain healthy blood vessels, and protect nerve tissue. Recent studies have identified a unique group of cells located on the surface of the brain that combine features of cells involved in lymphatic development with an exceptional ability to capture and process materials from the surrounding environment. Although these cells were first discovered in zebrafish, similar cell populations have since been reported in mammals, raising important questions about their identity and biological roles. In this review, we summarize current knowledge of how these cells develop, what makes them different from other blood vessel cells, how they help maintain the brain environment, and what is known about their possible involvement in disorders such as stroke and Alzheimer’s disease. We also discuss the latest technologies that are helping researchers better understand these cells and identify the key questions that remain unanswered. A better understanding of these specialized cells may improve our knowledge of brain health, blood vessel biology, and future strategies for preventing or treating neurological diseases.

## 1. Introduction

For decades, the central nervous system (CNS) was largely considered to lack a conventional lymphatic system and to possess only limited immune surveillance capacity. This view has changed substantially following the discovery of meningeal lymphatic vessels and glymphatic-associated clearance pathways, which have reshaped current understanding of cerebrospinal fluid circulation, immune regulation, and extracellular waste removal at CNS borders [1,2,3,4]. In parallel, advances in vascular biology and single-cell transcriptomics have revealed considerable endothelial heterogeneity within the CNS and its surrounding interfaces. These studies have drawn increasing attention to specialized endothelial-associated populations located between the meninges and cerebral vasculature. Among them, mural lymphatic endothelial cells (muLECs), also termed brain lymphatic endothelial cells (BLECs) in zebrafish, have emerged as an unusual non-lumenized endothelial population with distinctive developmental and functional properties. Related populations described in mammals, including fluorescent granular perithelial cells (FGPs), Mato cells, and leptomeningeal lymphatic endothelial-like cells (LLECs), share several morphological or molecular features, although their precise developmental and evolutionary relationships to zebrafish muLECs remain unresolved [5,6,7,8].

Early studies primarily emphasized the granular morphology and scavenger-like properties of mammalian perivascular cells, leading to their classification as macrophage-like populations [7,9,10]. This interpretation was reconsidered after zebrafish studies showed that BLECs/muLECs depend on Vegfc–Vegfr3 signaling and express lymphatic-associated markers [5,6]. These findings provide strong evidence for a developmental relationship between zebrafish muLECs and lymphatic endothelial programs. At the same time, their non-lumenized organization and pronounced endocytic activity distinguish them from conventional lymphatic endothelial cells [6,11,12]. Recent transcriptomic studies further support their classification as a specialized endothelial population [11,13], raising broader questions about the relationship between lineage identity and functional specialization at CNS borders.

Despite rapid progress, several major issues remain unresolved. Nomenclature remains inconsistent across studies and species, the developmental relationship between muLECs and conventional lymphatic endothelial cells is incompletely defined, and the molecular basis of their scavenger specialization remains poorly understood. In addition, it is still uncertain whether mammalian and human LLEC-like populations represent true homologues of zebrafish muLECs or instead reflect functionally convergent endothelial adaptations. A major conceptual limitation in the current literature is that developmental lineage and functional specialization are often treated as interchangeable, although these may represent partially independent dimensions of cell identity. In this review, we critically evaluate current evidence concerning the developmental origin, molecular identity, scavenger specialization, cross-species relationships, and potential pathological relevance of muLEC-related populations. We further discuss how muLECs provide a useful model for understanding endothelial heterogeneity and functional specialization at CNS borders, while clearly distinguishing established findings from unresolved evolutionary and mechanistic questions.

## 2. Literature Search Strategy

This Review was prepared as a narrative review based on a structured search of the literature. PubMed, Web of Science, and Google Scholar were searched using terms related to mural lymphatic endothelial cells, brain lymphatic endothelial cells, leptomeningeal lymphatic endothelial-like cells, fluorescent granular perithelial cells, Mato cells, scavenger endothelial cells, meningeal lymphatics, and CNS border endothelial populations. English-language articles available up to August 2026 were considered. Original studies were given priority, while review articles were mainly used to trace earlier work and provide broader context. Studies were included when they addressed the identity, development, localization, molecular features, scavenger function, or disease relevance of muLECs or closely related CNS border-associated populations. Papers dealing only with conventional meningeal lymphatic vessels were not included unless they were directly relevant to the questions discussed here. Reference lists of key papers were also checked for additional studies. As this is a narrative review, no formal risk-of-bias assessment or PRISMA-based study selection was performed.

## 3. Nomenclature and Cellular Identity of muLEC-Related Populations

The identification of muLECs has expanded current understanding of endothelial and perivascular cellular heterogeneity within the meningeal and cerebrovascular microenvironment. However, the terminology used to describe muLEC-related populations remains inconsistent and reflects differences in species, experimental approaches, and historical interpretation. In zebrafish, the terms brain lymphatic endothelial cells (BLECs) and mural lymphatic endothelial cells (muLECs) generally refer to the same vessel-associated endothelial population identified through developmental imaging and genetic analyses [5,6,7]. By contrast, fluorescent granular perithelial cells (FGPs) and Mato cells were initially described in mammals on the basis of their perivascular localization, autofluorescent granular morphology, and scavenger activity [7,14,15,16]. The more recent term leptomeningeal lymphatic endothelial-like cells (LLECs) has been used for mammalian leptomeningeal populations displaying partial molecular and structural similarities to zebrafish muLECs [8]. These terms should therefore not be treated as fully interchangeable without consideration of the underlying evidence. The main characteristics and current uncertainties of these muLEC-related populations are summarized in Table 1.

In zebrafish, several lines of evidence strongly support the endothelial and lymphatic-associated identity of BLECs/muLECs. These cells express endothelial and lymphatic-associated genes, including *lyve1*, *prox1*, and *flt4/vegfr3*, and their development is severely impaired by disruption of Vegfc, Ccbe1, or Vegfr3 signaling [5,6,17,18]. These findings distinguish zebrafish muLECs from conventional macrophage populations and establish a clear developmental relationship with the lymphatic endothelial lineage. More recent single-cell RNA sequencing studies have further identified muLECs as a transcriptionally distinct endothelial population enriched in lymphatic-associated programs together with genes involved in scavenger receptor activity, endocytosis, and intracellular cargo processing [11,13]. Collectively, these observations support the interpretation of zebrafish muLECs as a specialized endothelial population with lymphatic-associated developmental features.

Nevertheless, lymphatic lineage association does not necessarily imply equivalence to conventional lymphatic endothelial cells. Unlike classical LECs, muLECs do not generally form interconnected, lumenized vessels and instead remain individually or discontinuously distributed along the leptomeningeal surface in close association with cerebral blood vessels [11,12]. Their strong endocytic activity and enrichment of scavenger-related pathways further distinguish them from canonical fluid-draining lymphatic vessels. Thus, muLEC identity appears to reflect a combination of lymphatic-associated developmental programming and context-specific functional specialization at the CNS border.

**Figure 1:**
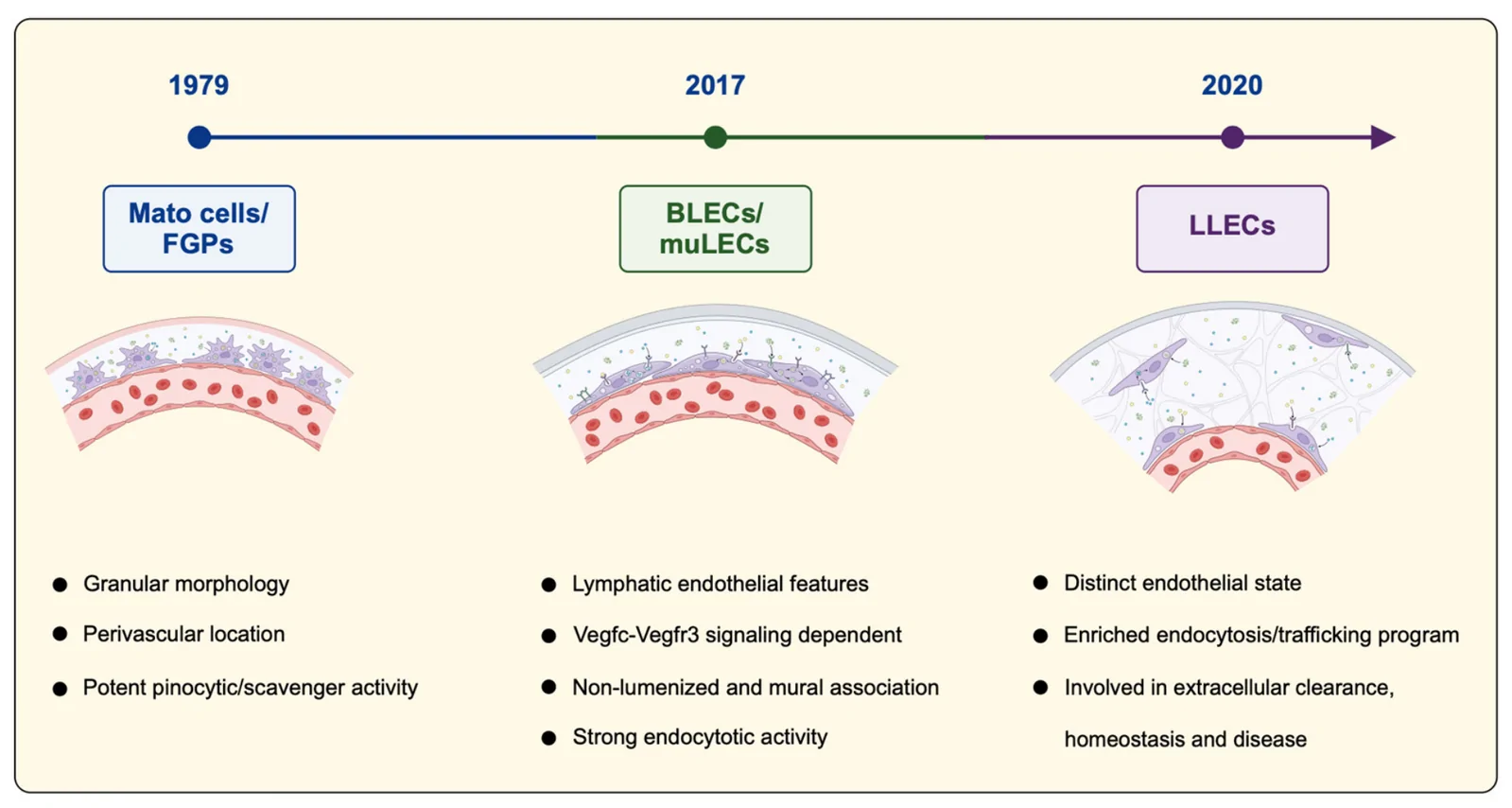
Historical evolution of muLEC nomenclature and biological characterization. The figure illustrates the transition from the initial description of Mato cells/fluorescent granular perithelial cells (FGPs) in 1979, to the identification of brain lymphatic endothelial cells (BLECs)/mural lymphatic endothelial cells (muLECs) in zebrafish in 2017, and subsequently to leptomeningeal lymphatic endothelial-like cells (LLECs) in mammals during the 2020s. Major advances include the recognition of lymphatic-associated molecular features, Vegfc–Vegfr3-dependent development, non-lumenized vascular association, and specialized endocytic and clearance-related functions. This timeline summarizes the historical evolution of terminology and characterization and does not imply a direct developmental or evolutionary sequence among these populations. Colored particles represent different extracellular materials, and arrows indicate their uptake by the illustrated cells. Colors are used for schematic distinction and do not represent specific molecular species.

The relationship between zebrafish muLECs and mammalian FGPs, Mato cells, or LLECs remains less certain. These populations share selected features, including perivascular or leptomeningeal localization, non-lumenized organization, and uptake of extracellular materials. However, direct evidence demonstrating equivalent developmental origins, conserved transcriptional states, or identical physiological functions remains incomplete. Mammalian populations may therefore represent true homologues, partially conserved cell states, or independently evolved endothelial adaptations with convergent scavenger functions. Current evidence supports grouping these cells as related CNS border-associated populations for comparative discussion, but not necessarily as a single universally conserved cell type. Accordingly, muLECs are best viewed as a specialized endothelial population whose classification requires consideration of developmental lineage, anatomical organization, molecular identity, and functional phenotype rather than any single defining marker or historical name (Figure 1).

## 4. Developmental Origin and Molecular Regulation of muLECs

The developmental origin of muLECs has become an important topic in cerebrovascular and lymphatic biology. Early zebrafish studies showed that these cells emerge during embryonic development and progressively distribute along the leptomeningeal surface in close association with cerebral blood vessels [5,6,7,19,20]. Development of BLECs/muLECs is highly dependent on Vegfc-Ccbe1-Vegfr3 signaling, consistent with the core molecular framework that regulates classical lymphangiogenesis [5,6,17,18]. Genetic disruption of *vegfc, ccbe1*, or *vegfr3/flt4* severely impairs the formation of these cells, supporting a developmental connection between muLECs and lymphatic endothelial programs. In parallel, muLECs express several transcription factors and markers associated with lymphatic endothelial specification, including *prox1*, *lyve1*, and *flt4/vegfr3* [5,6,21,22,23]. Together, these findings establish a developmental relationship between zebrafish muLECs and lymphatic endothelial programs while also highlighting several developmental and structural features that distinguish muLECs from conventional lymphatic endothelial cells.

Despite these developmental similarities, several developmental and structural features of muLECs remain atypical when compared with conventional lymphatic endothelial cells. Unlike classical meningeal lymphatic vessels, muLECs generally fail to form interconnected lumenized vascular structures and instead remain individually distributed along the leptomeningeal surface [11]. This distinction is notable because canonical lymphangiogenesis typically culminates in the formation of organized vascular networks specialized for fluid transport and immune trafficking [24,25,26]. Such an organization suggests that muLEC development diverges from the terminal differentiation process that normally produces classical lymphatic vascular networks. Although Vegfc-Vegfr3 signaling is clearly required for muLEC specification [5,6,17,18], the downstream mechanisms underlying this divergent developmental outcome remain incompletely understood.

Recent scRNA-seq analyses further expanded current understanding of muLEC molecular identity and developmental heterogeneity [11,13,27]. Several studies identified muLECs as a transcriptionally distinct endothelial population enriched for lymphatic-associated genes while simultaneously exhibiting molecular programs related to scavenger function, endocytosis, and intracellular trafficking, further distinguishing them from conventional lymphatic endothelial cells [11,13]. Trajectory analyses further raise the possibility that muLEC development may proceed through transient endothelial states rather than following a simple linear differentiation process. However, the developmental dynamics underlying muLEC maturation remain incompletely resolved, and it remains unclear whether muLECs arise from a relatively homogeneous precursor population or acquire distinct endothelial states during development. Resolving this question will be essential for determining whether endothelial heterogeneity within the muLEC population primarily reflects lineage diversification, functional specialization, or the integration of both processes.

Despite substantial progress in defining the signaling pathways involved in muLEC specification, several developmental questions remain unresolved. In particular, the precursor relationships, maturation dynamics, and mechanisms that prevent formation of conventional lumenized lymphatic structures remain poorly understood. Addressing these issues will require integrated lineage-tracing, live-imaging, and single-cell approaches together with spatial transcriptomics to resolve developmental transitions and functional specialization at high spatial and temporal resolution.

## 5. Functional Specialization of muLECs

One of the most distinctive biological features of muLECs is their specialized scavenger and endocytic activity. Zebrafish studies demonstrated that BLECs/muLECs efficiently internalize a broad range of extracellular substrates, including dextran, lipoproteins, protein-associated particles, and pathogen-derived materials [5,6,11,28]. Subsequent studies further showed that these cells possess high uptake capacity, in some experimental settings even exceeding that of microglia for specific tracers [12]. Morphologically, muLECs contain abundant intracellular vesicular and granular structures consistent with active endocytosis and intracellular trafficking [11,12,15,29]. In parallel, multiple scavenger-associated genes, including *mrc1a*, *stab1*, and *stab2*, are strongly enriched in these cells [11,13,30,31,32]. Together, these observations establish scavenger specialization as a defining functional feature of muLECs.

Despite increasing recognition of this scavenger phenotype, its mechanistic organization remains poorly understood. Most current evidence derives from fluorescent tracer uptake assays and static imaging analyses, whereas considerably less is known about how internalized cargo is sorted, processed, degraded, or recycled through intracellular trafficking pathways. Clarifying these processes will be important for determining whether muLECs function primarily as passive clearance cells or instead actively regulate the composition and homeostasis of the cerebrovascular extracellular environment.

The close anatomical association between muLECs and cerebral blood vessels further suggests that their scavenger activity may operate within a highly localized cerebrovascular context. Unlike classical lymphatic endothelial cells, which primarily function through organized lumenized drainage networks [24,33,34], muLECs remain individually distributed along the vascular surface. Several of these structural and functional features resemble specialized scavenger endothelial populations found in peripheral organs, particularly liver sinusoidal endothelial cells (LSECs) [35,36,37,38]. This comparison raises the possibility that muLECs represent a CNS border-associated endothelial specialization adapted for localized extracellular surveillance, cargo uptake, and molecular processing.

**Figure 2:**
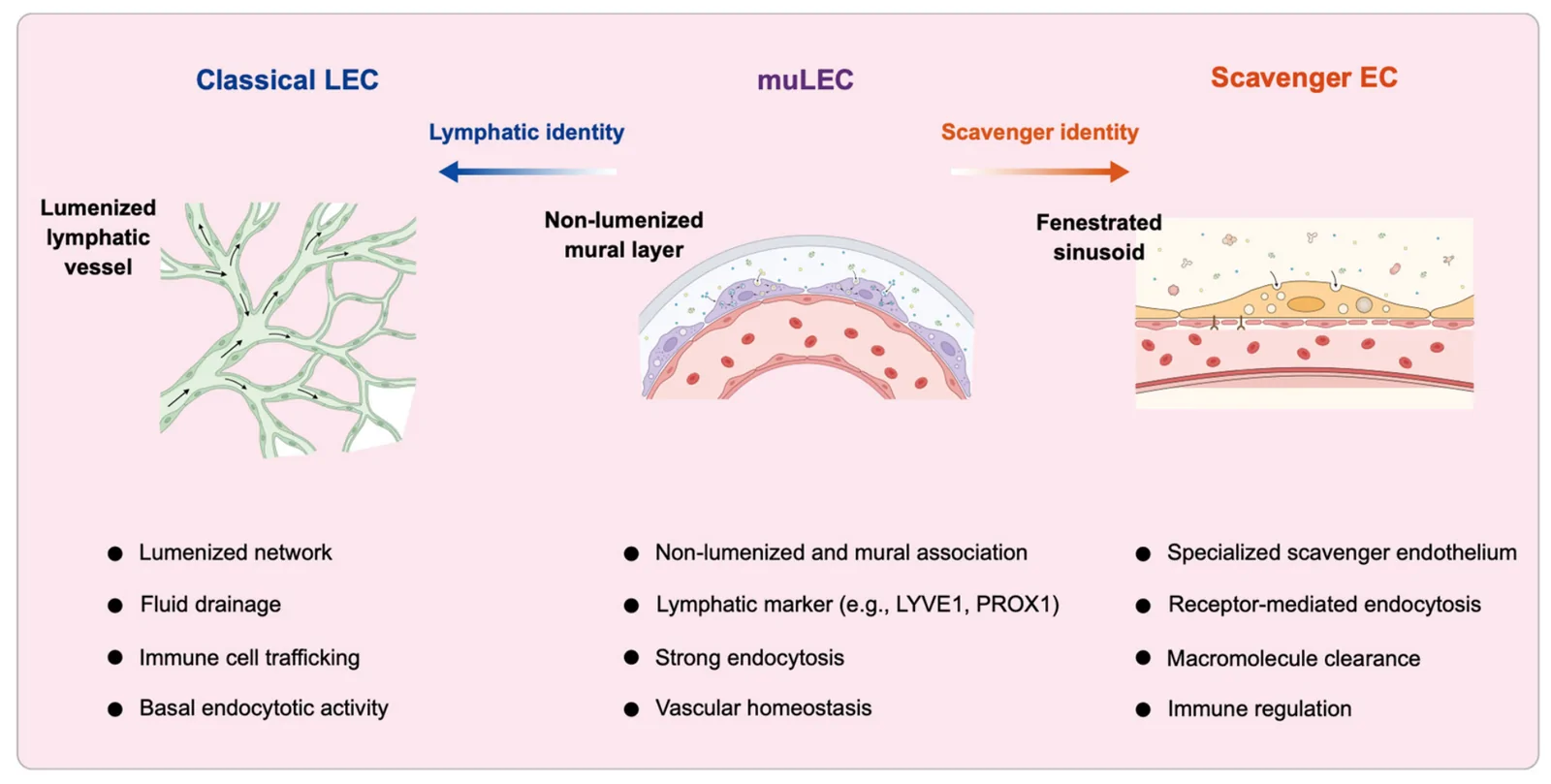
Conceptual framework of muLEC biology. The figure compares classical lymphatic endothelial cells (LECs), mural lymphatic endothelial cells (muLECs), and specialized scavenger endothelial cells (SECs). Classical LECs are characterized by lumenized vascular networks involved in fluid drainage and immune trafficking, whereas SECs are specialized for receptor-mediated uptake and macromolecular clearance. muLECs share developmental and molecular features with lymphatic endothelial cells while also displaying specialized endocytic properties. The model illustrates the hypothesis that muLECs may occupy a specialized cerebrovascular-associated endothelial state combining selected lymphatic-associated developmental features with scavenger and endocytic functions. The relationships illustrated are conceptual and should not be interpreted as experimentally established lineage or evolutionary relationships. Colors distinguish the different endothelial populations and anatomical components. Small colored particles represent extracellular materials involved in uptake and processing. Black arrows indicate lymphatic flow, whereas red arrows highlight the non-lumenized mural association of muLECs with the blood vessel.

Current studies provide limited insight into the extent and biological significance of functional heterogeneity within the muLEC population. It remains unclear whether all muLECs possess equivalent scavenger capacity, whether functionally distinct subpopulations exist, or how scavenger activity dynamically changes during development, aging, inflammation, and neurovascular injury. Addressing these questions will likely require integrated live imaging, functional genetics, and single-cell or spatial profiling approaches capable of linking molecular states with dynamic muLEC behavior across physiological and pathological conditions (Figure 2).

## 6. Cross-Species Relationships of muLEC-Related Populations

Most current knowledge regarding muLECs originates from zebrafish studies, where these cells can be directly visualized and genetically manipulated in vivo. The combination of optical transparency, vascular transgenic strategies, and live imaging accessibility has made zebrafish a particularly powerful system for investigating muLEC development and behavior within the intact cerebrovascular environment [39,40,41]. Early zebrafish studies established the developmental relationship between BLECs/muLECs and lymphatic endothelial programs while simultaneously revealing several structural and functional features that distinguish these cells from conventional lymphatic endothelial cells [5,6,17,18]. Collectively, these studies positioned zebrafish as the principal experimental system for defining the biological properties of muLECs.

Evidence for muLEC-like populations in mammals remains substantially more limited than in zebrafish. The identification of meningeal lymphatic vessels in mammals renewed interest in LYVE1-positive leptomeningeal endothelial-like populations displaying partial similarities to zebrafish muLECs. Unlike classical dural meningeal lymphatic vessels, these LLEC-like populations are generally observed as individually distributed cells associated with leptomeningeal and perivascular regions rather than organized lumenized drainage networks [2,3,8,42]. Morphological observations together with recent transcriptomic analyses suggest that subsets of these cells share molecular features associated with extracellular uptake, endocytosis, and intracellular processing. These similarities raise the possibility that conserved or convergently evolved CNS border-associated endothelial programs may exist across vertebrate species, although direct evidence for cellular homology remains limited.

Nevertheless, several important limitations remain. Direct developmental lineage-tracing evidence for mammalian LLEC-like populations is still lacking, and functional evidence in vivo remains comparatively sparse. In addition, zebrafish and mammals differ substantially in meningeal organization, cerebrospinal fluid circulation, immune compartmentalization, and cerebrovascular architecture [2,3,19,43,44]. These anatomical and physiological differences may influence the spatial distribution, developmental cues, and functional demands of CNS border-associated endothelial populations. As a result, morphologically or transcriptionally similar endothelial populations in zebrafish and mammals may not necessarily share the same developmental origin or physiological roles.

Recent scRNA-seq and spatial transcriptomic studies further expanded current understanding of cellular heterogeneity within the CNS border environment [45,46,47,48,49]. Several datasets identified endothelial- or lymphatic-associated populations with partially overlapping molecular signatures across species, suggesting that certain CNS border-associated endothelial programs may be more broadly distributed than previously appreciated. At the same time, transcriptomic similarity alone is insufficient to establish developmental equivalence or identical biological function [50,51]. Future comparative studies should therefore integrate developmental lineage, molecular identity, anatomical organization, and functional phenotype rather than relying on transcriptomic similarity alone when defining cross-species relationships. This issue may be particularly relevant for muLEC biology, where developmental lineage relationships and functional specialization are frequently interpreted together despite limited direct comparative evidence.

Resolving these questions will likely require integrated comparative approaches combining lineage tracing, developmental analyses, live imaging, spatial transcriptomics, and direct functional studies across species. Clarifying which features of muLEC biology are evolutionarily conserved, which represent convergent endothelial adaptations, and which are species-specific innovations will be essential for understanding endothelial specialization at CNS borders.

## 7. Potential Roles of muLECs in Neurological and Cerebrovascular Diseases

Increasing evidence suggests that dysfunction of CNS border-associated clearance systems contributes to the progression of neurological and cerebrovascular diseases [44,52,53,54]. Impaired handling of extracellular proteins, inflammatory mediators, and metabolic by-products has been increasingly linked to chronic inflammation, vascular dysfunction, and neurodegenerative progression [52,53,54,55,56]. Because muLECs localize along the interface between the meninges and cerebral vasculature and display unusually strong uptake capacity, these cells have attracted growing interest as potential regulators of CNS border homeostasis under pathological conditions [11]. However, direct experimental evidence supporting these proposed roles remains limited.

Neurodegeneration represents one of the major disease contexts in which CNS border-associated clearance pathways have attracted substantial attention. Accumulation of extracellular protein aggregates, including amyloid-β and tau-associated materials, is a central pathological feature of Alzheimer’s disease and related disorders [57,58,59,60]. The emergence of glymphatic and meningeal lymphatic research substantially reshaped current thinking regarding brain border-associated fluid exchange and waste handling in neurodegenerative disease [2,3,44,52,61]. Although zebrafish BLECs/muLECs display robust uptake of extracellular tracers and macromolecular substrates [5,11,12], direct evidence linking these cells to pathological protein regulation in vivo remains sparse. Whether muLECs actively contribute to neurodegenerative progression, facilitate protective clearance mechanisms, or primarily reflect adaptive endothelial responses to extracellular stress remains unresolved.

Apart from neurodegeneration, muLECs may also be relevant to cerebrovascular injury and inflammatory remodeling. Ischemic stroke, hemorrhage, and vascular damage all generate substantial accumulation of extracellular debris, blood-derived components, and inflammatory mediators within perivascular and meningeal regions [62,63,64,65]. Efficient handling of these materials is likely important for limiting secondary tissue injury and restoring vascular homeostasis [52,65]. Several zebrafish injury models further demonstrated that lymphatic endothelial-like cells participate in cerebrovascular regeneration and vascular remodeling following tissue damage [34,62,66]. Given their close association with the cerebrovascular surface, muLECs are well positioned to respond to dynamic changes in the extracellular environment surrounding injured vessels, potentially coupling local scavenger activity with broader neurovascular remodeling and inflammatory responses.

Despite increasing interest in these potential functions, direct mechanistic evidence linking muLECs to disease progression in vivo remains limited. Most available evidence comes from developmental mutants, tracer uptake assays, or acute injury models. These approaches are useful for defining cellular responses, but they do not readily distinguish whether muLECs actively modify disease processes or simply respond to changes in the local environment. Developmental perturbation may also introduce secondary vascular or meningeal changes that complicate interpretation at later disease stages. Much of the current work also remains focused on developmental biology or acute injury paradigms in zebrafish, whereas muLEC behavior during aging, chronic neurodegeneration, metabolic stress, or blood–brain barrier dysfunction remains poorly characterized. Similarly, disease-associated muLEC-like populations in mammals and humans remain insufficiently defined. Integrating comparative animal models with human spatial transcriptomic and pathological datasets will be important for evaluating the translational relevance of muLEC biology. Future studies using temporally controlled, cell-type-specific manipulation together with longitudinal imaging and functional analysis in mammalian models will be needed to determine whether muLECs directly influence pathological protein handling, inflammatory responses, vascular repair, or blood–brain barrier integrity. Determining whether muLECs actively contribute to disease pathogenesis, promote tissue repair, or primarily represent context-dependent endothelial adaptations will require direct functional investigation across diverse neurological and cerebrovascular disease models.

## 8. Emerging Technologies and Future Perspectives

Recent advances in imaging, single-cell analysis, and genetic manipulation have substantially expanded current investigation of cerebrovascular-associated lymphatic endothelial-like cells [19,47,48,67,68]. Among available experimental systems, zebrafish remain particularly valuable because their optical transparency enables direct visualization of muLEC behavior within the intact cerebrovascular environment [11,19,69]. These approaches have progressively shifted the field from largely descriptive anatomical observations toward molecular and functional analysis of CNS border-associated endothelial populations. In parallel, transcriptomic studies further support the classification of zebrafish muLECs as a transcriptionally distinct endothelial population with specialized molecular programs that distinguish them from conventional lymphatic endothelial cells [11,13].

Despite this progress, several major questions remain unresolved. Current studies still rely on molecular profiling and tracer uptake-based analyses, whereas the relationship between transcriptional state, intracellular processing, and dynamic endothelial behavior in vivo remains poorly understood. In addition, whether muLEC-related populations identified across species represent evolutionarily conserved endothelial lineages, convergent functional adaptations, or both remains uncertain. Addressing these issues will require experimental strategies capable of integrating developmental lineage, molecular identity, and dynamic cellular behavior across physiological and pathological conditions.

Several emerging approaches may help address these unresolved questions from complementary perspectives. Lineage-tracing strategies will be important for defining the developmental origin and precursor relationships of muLECs. Spatial transcriptomic and multi-omic approaches could further link molecular states with anatomical localization and local cellular interactions, while single-cell epigenomic profiling may help distinguish stable lineage-associated programs from transient or environmentally induced cell states. Intravital and high-resolution live imaging may provide direct information on muLEC dynamics, cargo uptake, and interactions with nearby blood vessels in vivo. In parallel, inducible and cell-type-specific genetic manipulation will be particularly useful for testing whether these cells have causal roles in vascular homeostasis, injury responses, and disease-related processes.

More broadly, muLEC biology highlights the increasing complexity of endothelial organization at CNS borders. Rather than functioning solely as passive structural components, endothelial populations within these regions may adopt context-specific properties shaped by local microenvironmental demands. Understanding how these specialized endothelial states emerge, are maintained, and interact with surrounding neurovascular systems will provide important insights into endothelial heterogeneity and functional specialization at CNS borders.

## 9. Conclusions

The study of muLECs has increasingly challenged conventional views of endothelial identity at CNS borders. Although these cells share developmental features with lymphatic endothelial programs, their persistent non-lumenized organization, close vascular association, and unusual uptake behavior distinguish them from conventional lymphatic endothelial cells and lumenized lymphatic vessels. Rather than fitting neatly into existing lineage-based classification frameworks, muLECs appear to combine lymphatic-associated developmental features with cerebrovascular localization and border-associated scavenger specialization. This complexity suggests that endothelial populations at CNS interfaces may possess substantially greater functional plasticity and context-dependent specialization than previously appreciated.

At the same time, many fundamental questions remain unresolved. The mechanisms that shape muLEC maturation, the extent of functional diversity within muLEC-related populations, and the evolutionary relationships between zebrafish and mammalian systems all remain incompletely understood. Clarifying how these specialized endothelial states emerge, adapt, and function across distinct CNS border environments may ultimately reshape current understanding of neurovascular organization, endothelial heterogeneity, and functional specialization in the brain. Functional studies in mammalian systems will be particularly important for determining whether muLEC-related populations share conserved physiological functions with zebrafish muLECs.

## Figures and Tables

**Table 1:** Comparison of muLEC-related populations reported across species.

Population	Species	Reported Markers/Features	Morphology	Anatomical Localization	Reported or Proposed Functions	Major Uncertainty
BLECs/muLECs	Zebrafish	*lyve1*, *prox1*, *flt4/vegfr3*; scavenger-associated genes including *mrc1a* and *stab2*	Non-lumenized, individually distributed endothelial cells	Leptomeningeal surface, closely associated with cerebral blood vessels	Uptake and processing of extracellular macromolecules; regulation of meningeal angiogenesis	Extent of functional heterogeneity and relationship to conventional lymphatic endothelial cells
FGPs	Rodents and other mammals	Historically defined mainly by autofluorescent granules and scavenger properties rather than a uniform molecular marker set	Granular, perivascular cells	Perivascular regions of the cerebral cortex	Uptake and clearance of extracellular macromolecular material	Developmental origin and relationship to endothelial or macrophage lineages remain incompletely resolved
Mato cells	Rodents	Historically characterized by fluorescent granules and perivascular localization	Granular perivascular cells	Around cerebral vessels, particularly arterioles	Proposed scavenger and barrier-associated functions	Whether Mato cells correspond directly to FGPs or other modernly defined CNS border populations remains uncertain
LLECs	Mammals	LYVE1-positive and partial lymphatic/endothelial-associated molecular features	Generally non-lumenized, individually distributed cells	Leptomeningeal and perivascular regions	Proposed extracellular uptake and local clearance-related functions	Direct lineage-tracing evidence and functional validation relative to zebrafish muLECs remain limited

## Data Availability

No new data were created or analyzed in this study. Data sharing is not applicable to this article.

## Abbreviations

The following abbreviations are used in this manuscript:

BBB	Blood–brain barrier
BLECs	Brain lymphatic endothelial cells
CNS	Central nervous system
FGPs	Fluorescent granular perithelial cells
LECs	Lymphatic endothelial cells
LLECs	Leptomeningeal lymphatic endothelial-like cells
LSECs	Liver sinusoidal endothelial cells
muLECs	Mural lymphatic endothelial cells
scRNA-seq	Single-cell RNA sequencing
